# SUMOylation Is Essential for Dengue Virus Replication and Transmission in the Mosquito *Aedes aegypti*

**DOI:** 10.3389/fmicb.2022.801284

**Published:** 2022-04-27

**Authors:** Shih-Che Weng, Shin-Hong Shiao

**Affiliations:** Department of Tropical Medicine and Parasitology, College of Medicine, National Taiwan University, Taipei, Taiwan

**Keywords:** *Aedes aegypti* (mosquito), dengue virus (DENV), envelop protein, SUMOylation, Ubc9

## Abstract

Small ubiquitin-like modifier (SUMO) is a reversible post-translational protein modifier. Protein SUMOylation regulates a wide variety of cellular processes and is important for controlling virus replication. Earlier studies suggest that dengue virus envelope protein interacts with Ubc9, the sole E2-conjugating enzyme required for protein SUMOylation in mammalian cells. However, little is known about the effect of protein SUMOylation on dengue virus replication in the major dengue vector, *Aedes aegypti*. Thus, in this study, we investigated the impact of protein SUMOylation on dengue virus replication in *A. aegypti*. The transcription of *A. aegypti* Ubc9 was significantly increased in the midgut after a normal blood meal. Silencing AaUbc9 resulted in significant inhibition of dengue virus NS1 protein production, viral genome transcription, and reduced viral titer in the mosquito saliva. In addition, we showed that dengue virus E proteins and prM proteins were SUMOylated post-infection. The amino acid residues K51 and K241 of dengue virus E protein were essential for protein SUMOylation. Taken together, our results reveal that protein SUMOylation contributes to dengue virus replication and transmission in the mosquito *A. aegypti*. This study introduces the possibility that protein SUMOylation is beneficial for virus replication and facilitates virus transmission from the mosquito.

## Introduction

Dengue virus infection causes several disease manifestations, ranging from dengue fever (DF) to life-threatening dengue hemorrhagic fever/dengue shock syndrome (DHF/DSS) (Wellekens et al., 2020; Roy and Bhattacharjee, 2021; Sharp et al., 2021). DF is one of the most significant arthropod-borne viral diseases, and is caused by four serotypes of DENV (Wilder-Smith et al., 2019; Roy and Bhattacharjee, 2021; Sharp et al., 2021). DENV is a positive-sense single-stranded RNA virus that belongs to the *Flaviviridae* family and is transmitted to humans primarily through the bites of infected mosquitoes of *Aedes* spp. (Wilder-Smith et al., 2019; Roy and Bhattacharjee, 2021; Sharp et al., 2021). Current studies indicate that more than 390 million DENV infections are reported worldwide annually (Bhatt et al., 2013; Shepard et al., 2016; Stanaway et al., 2016; Cattarino et al., 2020).

Transmission of DENV occurs via female *Aedes* mosquitoes, primarily *Aedes aegypti*, and, to a lesser extent, *Aedes albopictus*. Generally, the mosquitoes acquire the virus while taking a blood meal from an infected person (Wellekens et al., 2020; Roy and Bhattacharjee, 2021; Sharp et al., 2021). Within the mosquito, DENV initially infects and replicates in the mosquito midgut epithelial cells. Five to seven days post-infection (dpi), DENV disseminates to other tissues of the mosquito via the hemolymph. Upon arriving at the mosquito’s salivary gland, the virus undergoes further replication and becomes primed for transmission to another host (Salazar et al., 2007; Mukherjee et al., 2019).

Protein posttranslational modifications (PTMs) play essential roles in many cellular processes (Khoury et al., 2011; Silva et al., 2013; Loboda et al., 2019). Target proteins can be modified by adding small molecules (phosphorylation, glycosylation, acetylation, and methylation) and small proteins (ubiquitination, SUMOylation, and neddylation) (Khoury et al., 2011; Silva et al., 2013; Loboda et al., 2019). SUMOylation, as a PTM, is essential for various biological functions including cell growth, cell migration, cellular response to stress, and tumorigenesis (Yang et al., 2017; Loboda et al., 2019; Chang and Yeh, 2020). SUMO modification is well conserved between various species, including *Homo sapiens, Drosophila melanogaster*, and *Saccharomyces cerevisiae* (Epps and Tanda, 1998; Fraser et al., 2000; Apionishev et al., 2001; Hayashi et al., 2002; Li et al., 2021). Covalent linkages between lysine residues of target proteins and SUMO proteins are regulated by the hierarchical action of E1 SUMO activating enzyme complex SAE1/2, the E2 conjugating enzyme Ubc9, and E3 SUMO ligases, while deconjugation is performed by SUMO-specific proteases (Johnson, 2004; Loboda et al., 2019; Chang and Yeh, 2020; Li et al., 2021).

Changes in protein SUMOylation may occur following heat shock, DNA damage, proteasome inhibition, and other cellular stimuli, such as viral infection (Wimmer and Schreiner, 2015; Yang et al., 2017; Chang and Yeh, 2020; Li et al., 2021). During infection and replication, viruses manipulate the SUMOylation process to ensure viral persistence within the host (Varadaraj et al., 2014; Wimmer and Schreiner, 2015; Loboda et al., 2019; El Motiam et al., 2020). Multiple depletion studies have implicated components of the SUMOylation pathway in viral survival, pathogenesis, and host immunity (Chiu et al., 2007; Brown et al., 2016; Conn et al., 2016; Su et al., 2016; Guo et al., 2017; Feng et al., 2018; Zhu et al., 2019; Stokes et al., 2020).

Previous studies have shown that most eukaryotic organisms express both SUMO1 and a SUMO2/3 paralog. Insects, however, do not possess a SUMO1 paralog, and the SUMO2/3 paralogs lack the SUMO consensus motif (SCM) (Choy et al., 2013; Urena et al., 2016). This suggests that insect SUMO mechanisms lack the ability to efficiently form poly-SUMO chains without the presence of an E3 ligase, indicating an extra degree of regulation in insects. Additionally, studies have shown that *D. melanogaster* SUMO (DmSUMO) is not able to form poly-SUMO chains due to the lack of a SCM (Urena et al., 2016). A study of *A. aegypti* SUMO (AaSUMO) pathways showed poly-SUMO chains form more efficiently in the presence of an *A. aegypti* Protein Inhibitor of Activated STAT (AaPIAS). Depletion of AaSUMO, AaUbc9, or AaPIAS in *A. aegypti* cell lines resulted in a small but consistent significant increase in Zika virus (ZIKV) replication (Stokes et al., 2020). However, the *in vivo* roles of protein SUMOylation in regulating viral replication in the mosquito *A. aegypti* are yet to be elucidated.

Here, we demonstrate that protein SUMOylation is essential for dengue virus replication and transmission in the mosquito *A. aegypti*. Silencing of AaUbc9 resulted in significant inhibition of dengue virus replication in the mosquito. Moreover, we showed that dengue virus envelop (E) protein and pre-membrane (prM) protein were SUMOylated. Finally, our results revealed that amino acid residues K51 and K241 of dengue virus E protein are required for protein SUMOylation. Our findings reveal how viruses manipulate the SUMOylation process, as well as provide new targets for potential antiviral therapies.

## Materials and Methods

### Mosquitoes

Mosquitoes (*Ae. aegypti* UGAL/Rockefeller strain) were kept at 28°C and 70% relative humidity under a 12 h: 12 h light-dark cycle as previously described (Sri-In et al., 2019). Hatched larvae were transferred to plastic containers with sufficient water and fed yeast extract daily. Pupae were collected and transferred to a plastic container in an insect dorm. Emerged mosquitoes were fed using cotton balls soaked in 10% sucrose solution. Female mosquitoes were used for our experiments at 3–5 days post-eclosion (PE). The sucrose-soaked cotton balls were removed at least 12 h before blood-feeding. Female mosquitoes were permitted to blood-feed on an anesthetized Institute of Cancer research, ICR strain mouse for 15–30 min. ICR strain mice were anesthetized via an intraperitoneal injection of avertin at a dose of 0.2 ml/10 g. All animal procedures and experimental protocols were approved by an AAALAC-accredited facility and the Committee on the Ethics of Animal Experiments of the National Taiwan University College of Medicine (IACUC approval No: 20200210).

### Cell Culture and Virus

*Aedes albopictus* C6/36 and *Ae. aegypti* CCL-125 cell lines were cultured in Dulbecco’s Modified Eagle’s Medium (DMEM) and Mitsuhashi and Maramorosch Insect Medium (MM) in a 1:1 ratio containing 2% heat-inactivated fetal bovine serum and 1%penicillin–streptomycin solution. For virus production, C6/36 cells were infected with the DENV serotype 2 (DENV2) strain 16681 at a multiplicity of infection of 0.01. The culture supernatant was harvested on day 7 post-infection and stored at −80°C. To determine the viral titer, the virus stock was subjected to examination using a plaque assay, as previously described (Sri-In et al., 2019). Approximately 1.0 × 10^7^PFUs/mL DENV2 were used to infect the mosquitoes. For Ubc9 inhibitor (2-D08) assay, *Aedes aegypti* CCL-125 cells infected with DENV2 16681 strain at MOI of 1 for 2 h. Cells were incubated with 2-D08 before, simultaneously, or after DENV2 infection for 2 h. Intracellular DENV NS1 protein was measured by cell-based enzyme-linked immunoassay (ELISA).

### Oral Infection of Mosquitoes and Mosquito Saliva Collection

Infection of mosquitoes was achieved through an infectious blood meal via folded Parafilm-M. After starvation through sugar deprivation for 24 h, female mosquitoes were subsequently provided an infectious blood meal prepared by mixing 200 μl of mouse whole blood, 50 μl of 1 mM ATP, and 250 μl of DENV2 16681 strain (2.5 × 10^6^ PFU in 250 μl). After the blood feeding, each mosquito was examined on a stereo microscope to determine whether it had taken a full meal. Mosquitoes were kept at 28°C and 70% relative humidity under a 12 h:12 h light-dark cycle as previously described (Sri-In et al., 2019, 2020). To collect saliva, female mosquitoes were starved for 24 h prior to saliva collection. On the day of saliva collection, the feeding solution [ATP-containing phosphate-buffered saline (PBS)] was wrapped in stretched Parafilm-M membrane and put on the top of a container covered with nylon mesh, allowing mosquitoes to feed on the meal. The mosquito saliva-containing solution was removed from the membrane and transferred to a microtube and centrifuged at 12,000 × *g* for 1 min at 4°C. The protein concentration of mosquito saliva was measured using Bradford protein assays (Sri-In et al., 2019, 2020).

### RNA Extraction and Reverse Transcription

The whole bodies of 3–5 mosquitoes were collected in 1.5-ml tubes containing 0.5 ml of TRIzol (Invitrogen, Carlsbad, CA, United States). Tissue was homogenized using a rotor-stator homogenizer at room temperature for 5 min and centrifuged at 15,890 × *g* for 10 min at 4°C. After centrifugation, the supernatant was transferred to a new micro-tube containing 0.1 ml of chloroform (J. T. Baker) and mixed thoroughly at room temperature for 3 min. Samples were then centrifuged at 15,890 × *g* for 15 min at 4°C, and the supernatant was transferred carefully to a new micro-tube containing 0.25 ml of isopropanol (J. T. Baker). Samples were gently mixed and stored at −80°C for 30 min. After precipitation, the samples were again centrifuged at 15,890 × *g* for 30 min at 4°C. The supernatant was discarded, and 0.5 ml of 75% ethanol (Taiwan Burnett International Co., Ltd, Taipei, Taiwan) was used to wash the RNA pellet. All resulting samples were centrifuged at 15,890 × *g* for 5 min at 4°C, and the supernatant was discarded. Finally, the RNA pellet was dried in a laminar flow hood and dissolved in DEPC-H_2_O. After Baseline-ZEROTM DNase (Epicenter, Madison, WI, United States) treatment, the RNA sample was stored at −80°C. The RNA concentration was quantified using a spectrophotometer (Nanodrop 2000, Thermo Fisher Scientific, Waltham, MA, United States), and the sample was diluted with DEPC-H_2_O to a concentration of 1 μg/μl. The RNA samples were reverse-transcribed to cDNA using a High-Capacity cDNA Reverse Transcription Kit (Applied Biosystems, Waltham, MA, United States). The cDNA samples were stored at −20°C for further use. Gene expression was analyzed via real-time quantitative polymerase chain reaction (RT-qPCR). The ribosomal protein S7 gene was used as an internal control.

### Real-Time Quantitative PCR

The SYBR Green dye binding system was used for RT-qPCR in this study. SYBR Green binds the minor groove of DNA, and the target gene expression was quantified by detecting the resulting fluorescence signal. The cDNA sample was quantified using a KAPA SYBR FAST Universal qPCR kit (KAPA), via the qPCR primers (S7: 5′-TCAGTGTACAAGAAGCTGACCGGA-3′/5′-TTCCGCGCGCGCTCACTTATTAGATT-3′; DENV:5′-GAAGA CATTGACTGYTGGTGCAA-3′/5′-CGATGTTTCCACGCCCC TTC-3′). The PCR protocol consisted of initial denaturation at 95°C for 3 min, followed by 40 cycles of 3 s at 94°C and 40 s at 60°C. Fluorescence readings were measured at 72°C after each cycle. The target gene signal was detected and analyzed using the ABI 7900HT Fast Real-Time PCR System, and relative quantification results were normalized using the ribosomal protein S7 gene as an internal control.

### Double-Stranded RNA Preparation

RNA interference (RNAi) primers were designed using the E-RNAi webservice^1^. The T7 promoter sequence (5′-TAATACGACTCACTATAGGG-3′) was incorporated into all forward and reverse RNAi primers. The target gene fragment was amplified using Ex Taq DNA Polymerase (Takara, Kusatsu-shi, Japan). Fragments were amplified and cloned into a pCR 2.1-TOPO vector at 23°C for 30 min using a TOPO TA Cloning Kit (Invitrogen, Carlsbad CA, United States). The constructed plasmid was transformed into HIT-DH5α competent cells. Plasmids from positive colonies were purified using a FarvoPrep™ Plasmid DNA Extraction Mini Kit (Favorgen, Taipei, Taiwan) and sequenced to confirm that the cDNA was in frame.

The plasmid was digested by a restriction enzyme, and fragments were separated using 1% agarose gel. Target fragments were isolated and purified from the gel using a FarvoPrep™ GEL/PCR Purification Kit (Favorgen, Taipei, Taiwan). The fragments were then amplified using Ex Taq DNA Polymerase and purified using a FarvoPrep™ GEL/PCR Purification Kit. The purified PCR product was used as the template to synthesize Double-Stranded RNA (dsRNA) *in vitro* using a T7-Scribe™ Transcription Kit (Epicenter, Madison, WI, United States). The reaction was performed at 37°C for 4–12 h. A solution of 95 μl of DEPC-H_2_O and ammonium acetate (stop solution) was added to stop the reaction, and the supernatant was transferred into a new Eppendorf tube containing 150 μl of a phenol/chloroform (Amresco) solution. Samples were centrifuged at 15,890 × g for 5 min, at 4°C, and the supernatant was transferred to a new Eppendorf tube containing 150 μl of chloroform. After centrifugation at 15,890 × g for 5 min at 4°C, the supernatant was transferred to a new Eppendorf tube containing 110 μl of isopropanol. Samples were gently mixed and stored at −80°C for 30 min. Finally, each sample was centrifuged at 15,890 × *g* for 30 min at 4°C. The dsRNA pellets were dried in a laminar flow hood and dissolved in DEPC-H_2_O. The dsRNA was diluted to a final concentration of 5 μg/μl. Between day 3 and 5 PE, female mosquitoes were injected with 280 nl of dsRNA (5 μg/μl) using a Nanoject II AutoNanoliter Injecter (Drummond Scientific, Broomall, PA, United States). dsRNA against LacZ was used as the control dsRNA (dsLacZ) (Sri-In et al., 2019). Silencing efficiency was confirmed by collecting the total RNA of mosquitoes 3 days post-injection for RT-PCR.

### Western Blot Analysis

The whole bodies of three to five mosquitoes or the tissues of 30–50 mosquitoes were collected in 1.5-ml tubes containing 100 μl of protein lysis buffer [50 mM Tris (pH 7.4), 1% IGEPAL, 0.25% sodium deoxycholate, 150 mM NaCl, 1 mM EDTA, 1 mM phenylmethyl-sulfonylfluoride, 1X protease inhibitor mixture, 1X phosphatase inhibitor mixture, and 10 mM *N*-ethylmaleimide (Sigma-Aldrich, St. Louis, MO, United States)] and homogenized using a rotor-stator homogenizer. Each homogenized sample was centrifuged at 15,890 × *g* for 30 min at 4°C, and the supernatant was transferred to a QIAshredder™ column (Qiagen, Hilden, Germany). The eluted samples were collected and transferred to new Eppendorf tubes at −80°C. The protein concentration was quantified using the Bradford method and Bio-Rad Protein Assay Dye Reagent (Bio-Rad Laboratories, Inc, Hercules, CA, United States). Each protein sample was mixed with the same volume of sample buffer Laemmli 2 × Concentrate (Sigma-Aldrich, St. Louis, MO, United States) and adjusted to the same volume with 1 × sample buffer. To denature proteins for electrophoresis, protein samples were incubated at 65°C for 15 min. The protein samples (40 μg) were subjected to SDS-PAGE and blotted onto a PVDF membrane (Pall Corporation, New York, NY, United States) for 1.5 h. The membranes were blocked with 1% bovine serum albumin (BSA) in 1 × phosphate-buffered saline containing 0.4% Tween 20 (PBST) at room temperature for 1 h. Afterward, the membranes were incubated in PBST containing primary antibody overnight at 4°C. The antibodies used in this study were: mouse anti-SUMO antibody (Developmental Studies Hybridoma Bank, SUMO-2 8A2, 1/100), mouse anti-NS1 antibody (Yao-Hong Biotechnology, Taipei, Taiwan, YH0023, 1/10000), mouse anti-E antibody (Yao-Hong Biotechnology, Taipei, Taiwan, YH0026, 1/10000), mouse anti-prM antibody (from Dr. Kao-Jean Huang at the Development Center for Biotechnology, Taipei, Taiwan, 1/100), and rabbit anti-GAPDH antibody (GeneTex, Irvine CA, United States, GTX100118, 1/10000). Membranes were washed in PBST and incubated with secondary antibody [HRP-conjugated anti-mouse IgG (Abcam, Cambridge, United Kingdom, ab6728), or HRP-conjugated anti-rabbit IgG (GeneTex, Irvine CA, United States, GTX213110)] in PBST at room temperature for 1 h. Finally, membranes were washed in PBST and developed using WesternBright™ Peroxide and ECL (Advansta Inc. San Jose, CA, United States) as the substrate for horseradish peroxidase following the manufacturer’s instructions.

### Cell-Based Enzyme-Linked Immunoassay

The whole bodies and saliva of AaUbc9-silenced or dsLacZ-treated mosquitoes were collected in 100 μl of serum-free medium and stored at −80°C. C6/36 cells were seeded in a 96-well tissue culture plate and incubated at 28°C overnight. The homogenized suspensions of infectious mosquitoes were centrifuged at 18,928 × *g* for 30 min and kept on ice. The cell monolayers were washed with phosphate-buffered saline (PBS), and 50 μl of 10-fold serial dilutions of infectious mosquito suspensions were applied to the cells for 2 h. After virus absorption, each well was covered with 100 μl of culture medium, and the plates were incubated at 28°C for 4 days.

Cells were then fixed with 4% paraformaldehyde for 30 min at 4°C, washed four times with PBS, and treated with 0.1% Triton X-100 at room temperature for 1 h. The cells were subsequently incubated with blocking buffer (1% BSA, 0.5% Triton X-100 in PBS) at room temperature for 1 h. The blocking buffer was then substituted with 50 μl/well of a 1:1000 dilution of mouse monoclonal anti-NS1 IgG (Yao-Hong Biotechnology, Taipei, Taiwan) at 4°C overnight. After another four PBS washes, the cells were incubated with 30 μl/well of 1:500 dilution of HRP-conjugated anti-mouse IgG (Abcam, Cambridge, United Kingdom, ab6728) at room temperature. Two hours post antibody incubation, the cells were gently washed four times with PBS, followed by addition of 100 μl/well of 3,3′,5,5′-tetramethylbenzidine (TMB) substrate for HRP activity detection. The substrate reaction was inhibited using an equal volume of 1 N HCl. After reaction inhibition, the soluble yellow product that developed was read using a microtiter plate reader at a wavelength of 450 nm (Tan et al., 2014).

For cell viability assay, the 96-well tissue culture plates were stained with crystal violet staining solution (1% crystal violet in 75% ethanol) at room temperature for 30 min. After being washed four times in a stream of tap water, the plates were air-dried at room temperature. The stained cells were lysed in 1% SDS and measured the optical density of each well at 570 nm. Setting the OD570 of non-stimulated cells to 100%, and determine the percentage of 2-D08 treated cells that are viable by comparing the OD570 values of 2-D08 treated cells with the OD570 values of the non-stimulated cells (Feoktistova et al., 2016).

### Detection of Proteins SUMOylated *in vivo*

To identify targets of protein SUMOylation, we used a protocol applicable to a wide range of species (including *Homo sapiens* and *Mus musculus*, and other vertebrates such as *Gallus gallus*, *Xenopus laevis*, *Danio rerio*, and *D. melanogaster*) and applied it to SUMOylated protein identification in mosquitoes (Barysch et al., 2014). Briefly, the whole bodies of ten DENV2 infected mosquitoes were collected in 1.5-ml Eppendorf tubes containing 100 μl of 1X lysis buffer [1X PBS, 1% SDS, 5 mM EDTA, 1 mM phenylmethyl-sulfonylfluoride, 1X protease inhibitor mixture, 1X phosphatase inhibitor mixture, and 10 mM *N*-ethylmaleimide (Sigma-Aldrich, St. Louis, MO, United States)]. The solution was homogenized using a rotor-stator homogenizer and the resulting lysate was incubated at 65°C for 15 min. The lysate was then diluted with 900 μl of cold RIPA dilution buffer [1X PBS, 5 mM EDTA, 1 mM phenylmethyl-sulfonylfluoride, 1X protease inhibitor mixture, 1X phosphatase inhibitor mixture, and 10 μl of 10 mM *N*-ethylmaleimide (Sigma-Aldrich, St. Louis, MO, United States)] in a 10-fold dilution. The diluted lysate was centrifuged at 15,890 × *g* for 30 min at 4°C, and the supernatant was transferred to a QIAshredder™ column (Qiagen, Hilden, Germany). The eluted samples were collected and transferred to new Eppendorf tubes that were stored at −80°C. The protein concentration was quantified using the Bradford method and Bio-Rad Protein Assay Dye Reagent (Bio-Rad Laboratories, Inc, Hercules, CA, United States). Protein concentrations higher than 10 μg/μl were used as input material. We combined 1 ml of input material with antibody (1 μl of mouse anti-E, 100 μl of mouse anti-prM, 1 μl of mouse anti-NS1, or 100 μl of mouse anti-SUMO) and 10 μl of protein G-agarose beads, and incubated with gentle agitation at 4°C overnight. The following day, the antibody-coupled beads were centrifuged at 700 × *g* for 5 min at 4°C and the supernatant was removed. The antibody-coupled beads were washed three times in 1 ml of RIPA buffer with centrifugation after each wash at 700 × *g* and at 4°C for 5 min. The protein was eluted by adding 50 μl of 2X Laemmli sample buffer (Sigma-Aldrich, St. Louis, MO, United States) before incubation at 65°C for 15 min. Western blot was performed on the eluted samples to detect SUMO-modified proteins.

### Detection of Proteins SUMOylated *in vitro*

An *in vitro* SUMOylation assay kit (Abcam, Cambridge, United Kingdom, ab139470) was used to generate SUMOylated proteins *in vitro* via covalent isopeptide linkage of the c-terminus of SUMO protein to specific lysine residues on the target protein through the SUMOylation enzyme cascade. Target proteins (DENV2 or Zika virus), SUMO protein, SUMOylation enzyme (E1 and E2 of protein SUMOylation), SUMOylation buffer, and Mg-ATP were combined as per Abcam SUMOylation kit protocol and incubated at 37°C for 2 h to generate the SUMOylated product *in vitro*. A western blot was used to detect viral protein SUMOylation in the reaction mixture via antibodies against E protein, prM protein, and NS1 protein. The reaction mixture, omitting Mg-ATP cofactors (required for E1 activation), was used as a negative control.

## Results

### Protein SUMOylation Plays an Essential Role in Dengue Virus Replication in the Mosquito

**FIGURE 1:**
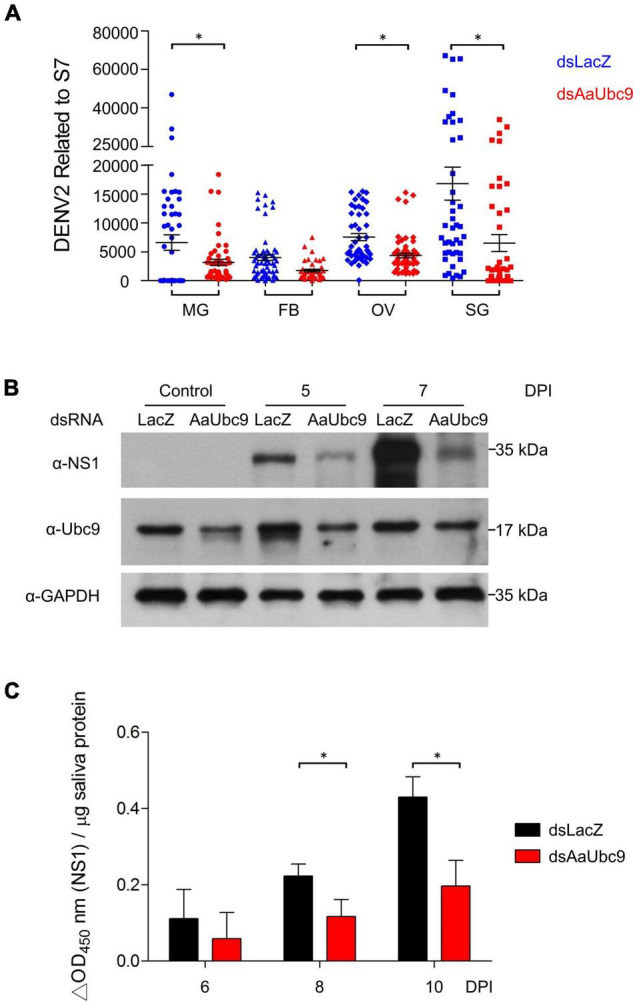
AaUbc9 is essential for dengue virus replication and infectivity. **(A)** Three-day-old female mosquitoes were injected with dsRNA of LacZ (dsLacZ) or AaUbc9 (dsUbc9) 3 days prior to DENV2 infectious blood feeding (10^7^ pfu/ml). Ten days post blood feeding, the midgut, body fat, ovary, and salivary gland were dissected, and total RNA was collected. The DENV2 genome transcription level was quantified using real-time PCR. The reactions were normalized to the ribosomal protein gene *s7*. Comparisons were performed using the Tukey’s multiple comparison test with dsLacZ injection group; *p ≤ 0.05. **(B)** Three-day-old female mosquitoes were injected with dsLacZ or dsAaUbc9. Three days post dsRNA injection the mosquitoes were infected with DENV2 through thoracic injection (200 pfu/mosquito). The protein levels of DENV2 NS1 were determined in female mosquitoes at 0, 5, and 7 days post viral injection by western blot with anti-NS1 antibody. GAPDH was used as an internal control. **(C)** Three-day-old female mosquitoes were injected with dsLacZ or dsAaUbc9 3 days prior to DENV2 infectious blood feeding (10^7^ pfu/ml). The level of DENV NS1 protein was measured in mosquito saliva 6, 8, and 10 days post blood feeding using cell-based ELISA with an anti-NS1 antibody. Comparisons were performed using the Tukey’s multiple comparison test with dsLacZ injection group; *p ≤ 0.05.

To investigate the roles of protein SUMOylation in virus replication in *A. aegypti*, we used a reverse genetic approach by introducing double-stranded RNA of AaUbc9 (dsAaUbc9) into the mosquito to silence AaUbc9 expression. Three-day-old female mosquitoes were injected with dsRNA of LacZ (dsLacZ) or dsAaUbc9 3 days prior to an infectious blood-feeding with DENV2 (10^7^ pfu/ml). Total RNA from the midgut, fat body, ovary, and salivary gland from individual female mosquitoes was collected 10 days post-infection. The expression of the DENV2 viral genome was quantified by RT-qPCR. Our results showed that in response to the depletion of AaUbc9, the expression of the viral genome was inhibited in the mosquito midgut, ovary, and salivary gland at 10 days post infection (Figure 1A). Next, we examined the effect of AaUbc9 silencing on viral protein production in mosquitoes. Female mosquitoes were injected with dsLacZ or dsAaUbc9 at 3 days prior to DENV2 virus infection by thoracic injection (200 pfu/mosquito). Total protein was collected at 5 and 7 days post-infection and the production of DENV2 virus NS1 protein was analyzed by western blot using an anti-NS1 antibody. The production of NS1 protein was inhibited by AaUbc9 silencing in mosquitoes (Figure 1B). The anti-Ubc9 antibody was used to validate the silencing of Ubc9 in mosquitoes. To further investigate the effect of protein SUMOylation on virus transmission, female mosquitoes were injected with dsLacZ or dsAaUbc9 3 days prior to DENV2 infection *per os* (10^7^ pfu/ml). Mosquito saliva was collected at 6, 8, and 10 days post-infection and the expression of infectious virus particles in saliva was determined by cell-based ELISA with an anti-NS1 antibody. The silencing of AaUbc9 significantly inhibited the expression of infectious virus particles in the saliva of mosquitoes (Figure 1C). Taken together, our results suggest that AaUbc9 regulates dengue virus replication and transmission in the mosquito *A. aegypti*.

### Ubc9 Inhibitor Blocks Viral Protein Production Post Virus Entry

**FIGURE 2:**
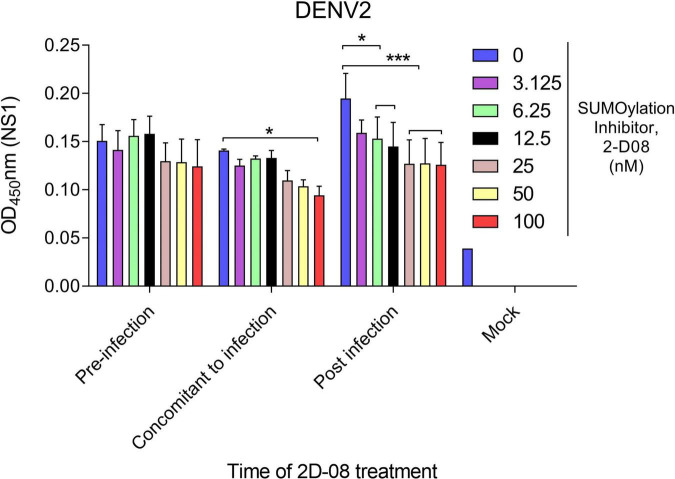
Ubc9 inhibition blocks viral replication post-entry. Time-of-drug administration experiments with Ubc9 inhibitor 2-D08. *A. aegypti* CCL-125 cells infected with DENV2 at MOI 1 were treated with inhibitor as indicated. Intracellular DENV NS1 protein was measured by cell-based ELISA. Comparisons were performed using the Tukey’s multiple comparison test with DMSO control group (0 nM); *p ≤ 0.05, ***p < 0.001.

Next, we made use of a Ubc9 inhibitor (2-D08) to investigate the interaction between protein SUMOylation and viral protein production. The cytotoxicity of Ubc9 inhibitor (2-D08) in *A. aegypti* CCL-125 cells were examined by cell viability assay (Supplementary Figure 1). The expression of DENV2 NS1 protein was analyzed by cell-based ELISA using an anti-NS1 antibody. Briefly, *A. aegypti* CCL-125 cells were treated with Ubc9 inhibitor at a variety of concentrations either before, simultaneously, or after DENV2 infection at a multiplicity of infection (MOI) of 1. Viral protein production was determined using a microtiter plate reader utilizing a mouse monoclonal anti-NS1 antibody followed by an HRP-conjugated anti-mouse antibody. Our data revealed that viral protein production was inhibited in response to the reduced AaUbc9 activity (Figure 2). It is worth noting that AaUbc9-mediated viral protein suppression is regulated at a stage post viral entry.

### Protein SUMOylation Pathway Is Activated After Virus Infection in the Mosquito

**FIGURE 3:**
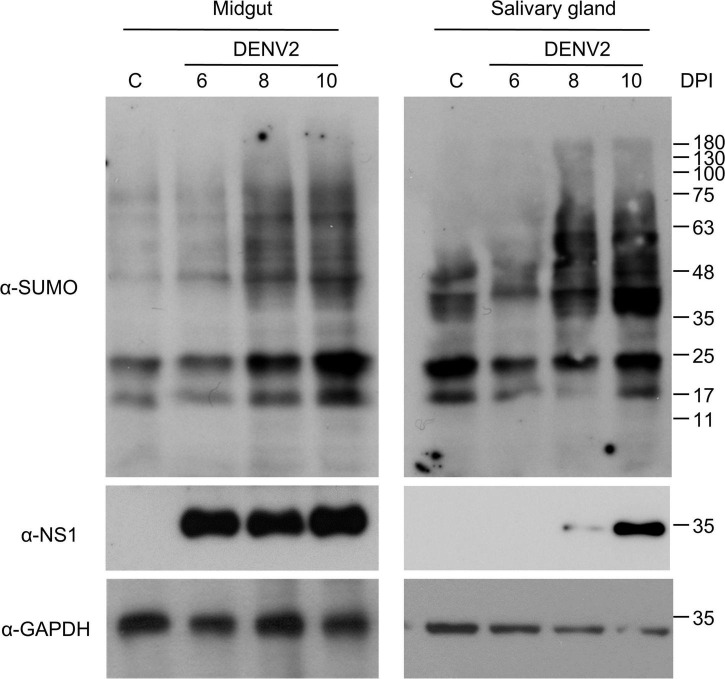
Protein SUMOylation is activated after a blood meal in the midgut of mosquitoes. Three-day-old female mosquitoes were fed with DENV2 infectious blood meal (10^7^ pfu/ml). At 6, 8, or 10 days post blood feeding the midgut and salivary gland were dissected, and the protein levels of SUMO and DENV2 NS1 protein were analyzed by western blot with anti-SUMO or anti-NS1 antibodies. GAPDH was used as an internal control.

When the mosquito take an infectious blood meal, the DENV first infects the epithelial cells of midgut. Five to seven days post-infection, DENV disseminates to other tissues of the mosquito via the hemolymph (Salazar et al., 2007; Mukherjee et al., 2019). To examine protein SUMOylation in the mosquito in response to dengue virus infection, total protein from mosquito midgut and salivary gland was collected at 6, 8, and 10 days post DENV2 infection. The expression of SUMO and NS1 proteins were determined by western blot analysis using anti-SUMO and anti-NS1 antibodies, respectively. Our results showed that protein SUMOylation was strongly activated at 8 and 10 days in midgut and salivary gland post DENV2 infection (Figure 3).

These results suggest that protein SUMOylation is induced after an infectious blood meal in the mosquito.

### Dengue Virus Envelope Protein and Pre-membrane Protein Are Potential Targets of Protein SUMOylation

**FIGURE 4:**
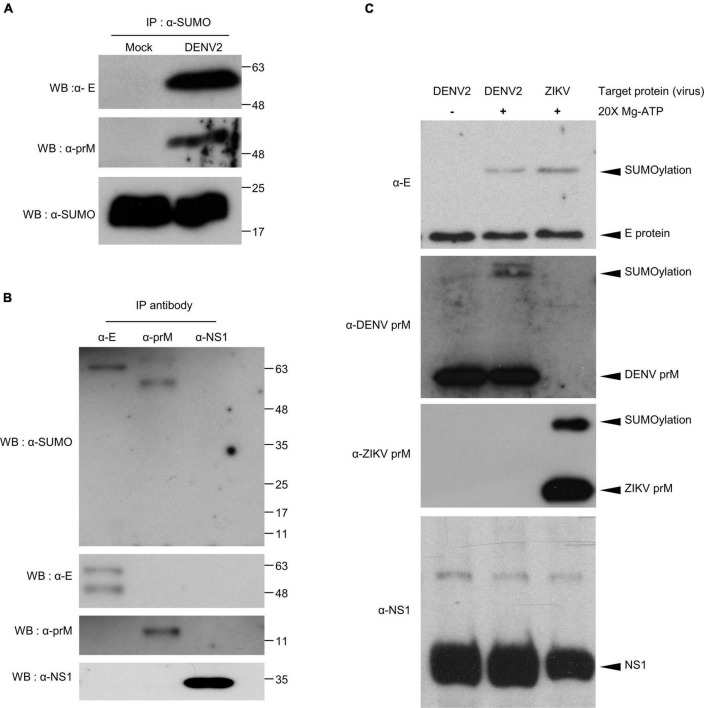
Covalent modification of DENV2 and ZIKV E and prM proteins by SUMO protein in virus-infected mosquitoes. **(A,B)** Seven days post virus injection (2000 pfu/mosquito), mosquito whole body cell lysate was collected and incubated at 65°C for 15 min. The denatured proteins were immunoprecipitated using antibodies specific to SUMO protein **(A)** and DENV proteins E, prM, and NS1 **(B)**. The immunoprecipitation products were analyzed via western blot with anti-SUMO, anti-E, anti-prM, and anti-NS1 antibodies. **(C)** *In vitro* SUMOylation assay of DENV proteins, E, prM, and NS1. The medium from DENV infected C6/36 cells was collected and examined via our *in vitro* SUMOylation assay. We used a western blot to probe the reaction mixture with anti-E, anti-prM, and anti-NS1 antibodies. A reaction without ATP was used as a negative control.

To further understand how SUMOylation influence DENV infection, it was necessary to identify specific target proteins by performing *in vivo* and *in vitro* protein SUMOylation assays. For the *in vivo* protein SUMOylation assay, we collected whole body protein from DENV2-infected mosquitoes. Input protein concentrations of at least 10 μg/μl were denatured and used in the immunoprecipitation reactions. The resultant data indicated dengue virus E and prM were potential target proteins for SUMOylation (Figures 4A,B).

To verify this observation, we proceeded with an *in vitro* protein SUMOylation analysis. It has been previously demonstrated that SUMO modification occurs through an ATP-dependent enzymatic cascade. The E1 enzyme, as an integral protein in this process, covalently links SUMO proteins through cysteine at the active site (Johnson, 2004; Chang and Yeh, 2020). Understanding this, we collected the medium from C6/36 cells infected with DENV2 or ZIKV. SUMO protein and SUMOylation enzymes (E1 and E2) were mixed with DENV- or ZIKV-containing medium and incubated to generate SUMOylated products *in vitro*. The reaction mixture was analyzed via western blot to examine SUMOylation of viral proteins (Figure 4C). The same reaction without ATP served as a negative control. In concert with our *in vivo* studies, this data suggests that the envelope proteins and pre-membrane proteins, but not non-structural protein 1 (NS1), of DENV2 and ZIKV are potential targets for protein SUMOylation.

### Dengue Virus Envelope Protein SUMOylation Is Essential for Virus Particle Production

**FIGURE 5:**
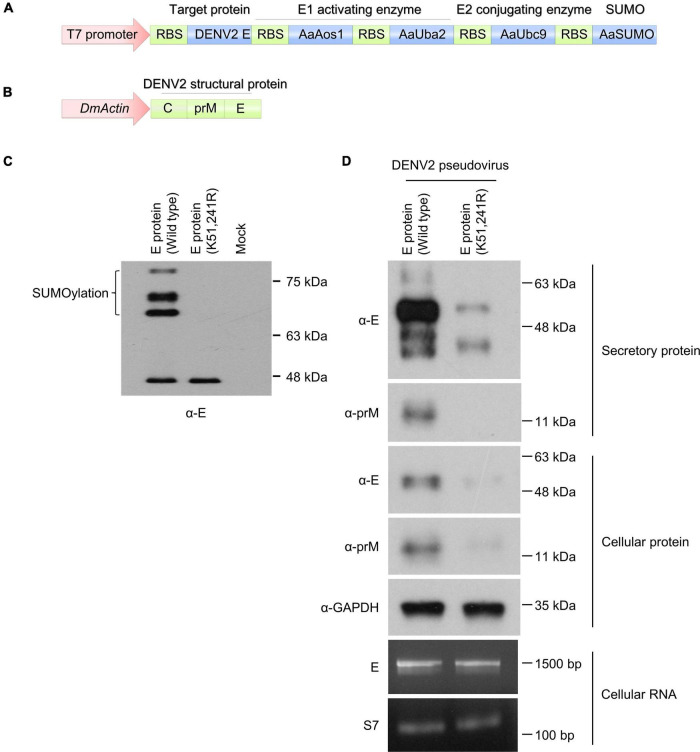
DENV2 envelope protein SUMOylation is essential for viral particle production in mosquito cells. **(A)** A plasmid encoding the linear fusion of mosquito SUMO-E1 (AaAos1 and AaUba2), SUMO-E2 (AaUbc9), SUMO protein, and target protein (wild type or K51/241R mutant of DENV2E). **(B)** A plasmid encoding the linear fusion of DENV2 prM and E proteins linked to an actin 5C promoter. **(C)** The plasmid depicted in A was transformed into *E. coli* BL21 (DE3) and protein expression was induced with 1 mM IPTG at 25°C. Four hours post protein transcription induction, the bacterial lysate was analyzed by western blot using anti-E antibody. **(D)** The plasmid described in B was transfected into CCL-125 cells. Four days post transfection the secretory virus-like particles, cellular protein lysate, and cellular RNA were collected and analyzed by western blot and RT-PCR.

To investigate and elucidate the role of dengue virus E protein SUMOylation in virus replication in mosquitoes, we blocked protein SUMOylation via site-directed mutagenesis. A previous study showed that the N-terminus of DENV2 non-structural protein 5 (NS5) was SUMOylated, leading to increased viral protein stability. However, the modification sites were unknown (Su et al., 2016). In contrast, other findings suggested that dengue virus E protein residues K51 and K241 are required for interaction with Ubc9 in mammalian cells, and that this interaction is essential for dengue virus E protein SUMOylation (Chiu et al., 2007). To investigate this issue, we constructed a plasmid containing the linear fusion of mosquito SUMO-E1 (AaAos1 and AaUba2), SUMO-E2 (AaUbc9), SUMO protein, and target protein variants (wild type and K51/241R mutant of DENV2 envelope protein), with transcription being controlled by a T7 promoter (Figure 5A). Prior to *in vivo* experimentation, SUMOylation was established in *Escherichia coli*. After protein synthesis induction with IPTG, the bacterial pellet was collected and analyzed by western blot to determine DENV2 E SUMOylation. Our results showed that the high molecular weight form of DENV2 E was only observed in samples with the wild type envelope protein but not those with the K51/241R mutant (Figure 5C). This indicates that the DENV2 envelope protein can be SUMOylated, and the amino acid residues K51 and K241 of dengue virus E protein are essential for protein SUMOylation.

We next performed site-directed mutagenesis to generate wild type and K51/241R mutant envelope protein containing pseudovirus particles. A plasmid containing the structural proteins [capsid (C), envelope (E), and membrane (M) proteins] of DENV2 strain 16681 was linked to the actin promoter of *D. melanogaster* (Figure 5B). The structural proteins of the DENV2 were expressed in the *A. aegypti* cell line CCL-125, leading to pseudoviral particle secretion. The supernatant of the transfected CCL-125 culture medium was collected and examined by western blot analysis. Secretion of E and prM proteins was suppressed in the K51/241R mutant expressing cells (Figure 5D, secretory protein), suggesting the inhibition of viral structural proteins secretion. The cellular protein of transfected CCL-125 from wild type and K51/241R mutant cells was then collected and examined by western blot analysis. Our results showed that the production of E and prM proteins from K51/241R mutants were inhibited (Figure 5D, cellular protein). Interestingly, the transcriptional expression of DENV2E protein in wild type and K51/241R mutant remained equal (Figure 5D, cellular RNA), suggesting the inhibition of E and prM proteins production from K51/241R mutant is transcription independent. Taken together, our results indicate that envelope protein SUMOylation is essential for viral structural proteins secretion, a critical step for virus transmission.

## Discussion

SUMO modification is known to be well-conserved between species; including *H. sapiens, D. melanogaster*, and *S. cerevisiae* (Epps and Tanda, 1998; Fraser et al., 2000; Apionishev et al., 2001; Hayashi et al., 2002; Urena et al., 2016; Li et al., 2021). Bioinformatic analysis of the amino acid sequence and tertiary structure of SUMO proteins, E2 conjugating enzyme (Ubc9), and SUMOylation activating heterodimer (SAE1/2) has demonstrated these high conservation rates (Stokes et al., 2020; Li et al., 2021). Interestingly, while previous studies show most eukaryotic organisms express both a SUMO1 and SUMO2/3 paralog, insects do not possess a SUMO1 paralog, and the insect SUMO2/3 paralogs lack the SCM (Choy et al., 2013; Urena et al., 2016). DmSUMO, as an example of this phenomenon, lacks a SCM and is thus unable to form poly-SUMO chains (Urena et al., 2016). This suggests that insect SUMO cannot efficiently form poly-SUMO chains without the presence of an E3 ligase. Although the amino acid sequence and predicted tertiary structure of AaSUMO is well conserved in *H. sapiens* SUMO3 (HsSUMO3), the lack of SCM in the N-terminus suggests that the biochemical function of AaSUMO is more similar to that of HsSUMO1 than HsSUMO3 (Stokes et al., 2020).

Poly-SUMO chain formation is important for many cellular processes across a range of species (Epps and Tanda, 1998; Fraser et al., 2000; Apionishev et al., 2001; Hayashi et al., 2002; Stokes et al., 2020). Regarding *A. aegypti*, AaSUMO can form poly-SUMO chains efficiently when in the presence of an AaPIAS (Stokes et al., 2020). The highly conserved formation of poly-SUMO chains across species, as well as the formation of SUMO conjugated proteins in *A. aegypti* (Figure 3), indicates that protein SUMOylation likely serves an important biological function in the mosquito *A. aegypti*.

Many studies have implicated components of the SUMOylation pathway in regulating viral survival, pathogenesis, and host immunity (Brown et al., 2016; Conn et al., 2016; Su et al., 2016; Feng et al., 2018; Stokes et al., 2020). It remains difficult to determine if a phenotype is directly or indirectly linked to the SUMOylation pathway because current studies are not able to distinguish between different targets of SUMO modification (Lyst and Stancheva, 2007). To further investigate the effect of protein SUMOylation on virus replication in mosquitoes and examine the interaction between protein SUMOylation and dengue virus replication, we utilized dsRNA to silence the expression of the SUMO-conjugating enzyme AaUbc9. Our data showed that production of the DENV genome, viral proteins, and infectious virus particles were inhibited in response to silencing AaUbc9 (Figure 1). Moreover, we showed that a Ubc9 inhibitor blocked virus production post viral entry (Figure 2). Taken together, our results indicate that AaUbc9 plays a key role in dengue virus replication in the mosquito.

In a previous study, depletion of AaSUMO, AaUbc9, or AaPIAS *in vitro* resulted in a small but consistent increase in ZIKV replication (Stokes et al., 2020). The authors proposed that this could be due to the modification of viral NS5 proteins or immune-related proteins, which normally function to suppress ZIKV replication. It remains difficult to distinguish the function of protein SUMOylation in mosquitoes, especially as they may be virus-specific in certain mosquito cell types or tissues. In addition, it is difficult to determine if a phenotype is directly or indirectly linked to the SUMOylation pathway. Therefore, we aimed to determine the target proteins of SUMOylation in mosquitoes post DENV infection.

Previously, dengue virus E and NS5 proteins have been identified as potential targets for protein SUMOylation in mammalian cells (Chiu et al., 2007; Su et al., 2016). Although specific lysine residues were not identified, DENV SUMO modification was noted at the N-terminus of NS5, increasing protein stability. Consequently, it was hypothesized that SUMO “floats” around the N-terminus of the protein and can modify any available lysine residues (Su et al., 2016). In this study, our data indicate that dengue virus E and prM proteins, but not NS1, are potential target proteins of SUMOylation. Paralleling this, ZIKV E and prM proteins were also identified as potential targets of SUMOylation (Figure 4).

A range of viral structural proteins are known to be SUMO modified, including HIV-1 p6 protein (Gurer et al., 2005), dengue virus E protein (Chiu et al., 2007), and coronavirus nucleocapsid protein (Li et al., 2005, 2006). A previous study demonstrated that dengue virus E protein interacts with Ubc9 through residues K51 and K241 in mammalian cells, and that this interaction plays a key role in dengue virus E protein SUMOylation (Chiu et al., 2007). To investigate this issue in mosquitoes, we developed an *in vitro* SUMOylation system in *E. coli.* Our results showed that dengue virus E protein is a target protein for protein SUMOylation, and residues K51 and K241 of the protein are crucial for protein SUMOylation in mosquitoes (Figure 5C). We further performed site-directed mutagenesis to generate wild type and K51/241R mutant envelope protein containing pseudovirus particles. Our results showed that E and prM proteins secretion and protein production were inhibited from K51/241R mutant (Figure 5D, secretory and cellular protein), which are important for viral transmission. However, the transcriptional expression of DENV2 E protein in wild type and K51/241R mutant remained equal (Figure 5D, cellular RNA), suggesting the inhibition of E and prM proteins production from K51/241R mutant is transcription independent. Additionally, in the pseudovirus system, the coding sequences of wild type and K51/241R mutant are both linked to the actin promoter of *D. melanogaster*. Therefore, the regulation of wild type and K51/241R mutant protein expression remained equal. These results raise the possibility that the inhibition of E and prM proteins production from K51/241R mutant are due to the regulation of protein stability. In conclusion, we demonstrated that protein SUMOylation is essential for dengue virus replication and transmission in the mosquito *A. aegypti*. Silencing of AaUbc9 resulted in significant inhibition of dengue virus replication in the mosquito. Moreover, we showed that dengue virus E and prM proteins were SUMOylated. Finally, our results revealed that amino acid residues K51 and K241 of dengue virus E protein are essential for protein SUMOylation. Our findings elucidate how the protein SUMOylation process manipulate virus replication, as well as provide new targets for potential antiviral therapies.

## Data Availability Statement

The original contributions presented in the study are included in the article/Supplementary Material, further inquiries can be directed to the corresponding author/s.

## Ethics Statement

The animal study was reviewed and approved by AAALAC-accredited facility and the Committee on the Ethics of Animal Experiments of the National Taiwan University College of Medicine (IACUC approval No: 20200210).

## Author Contributions

S-CW and S-HS contributed to conception and design of the study, performed the experiments and statistical analysis, and wrote the manuscript. Both authors contributed to manuscript revision, read, and approved the submitted version.

## Conflict of Interest

The authors declare that the research was conducted in the absence of any commercial or financial relationships that could be construed as a potential conflict of interest.

## Publisher’s Note

All claims expressed in this article are solely those of the authors and do not necessarily represent those of their affiliated organizations, or those of the publisher, the editors and the reviewers. Any product that may be evaluated in this article, or claim that may be made by its manufacturer, is not guaranteed or endorsed by the publisher.

## Footnotes

1
http://www.dkfz.de/signaling/e-rnai3//

## Supplementary Material

Supplementary Figure 1 Cytotoxic effect of Ubc9 inhibitor (2-D08) in *A. aegypti* CCL-125 cells. *A. aegypti* CCL-125 cells were incubated with culture medium containing Ubc9 inhibitor (2-D08) as indicated for 2 days. Attached cells were measured by cell viability assay.
