# Supplementary figures and images for: SUMOylation Is Essential for Dengue Virus Replication and Transmission in the Mosquito Aedes aegypti
Source: Front Microbiol. 2022 Apr 27;13:801284. doi: 10.3389/fmicb.2022.801284 (PMC9093690; doi:10.3389/fmicb.2022.801284)

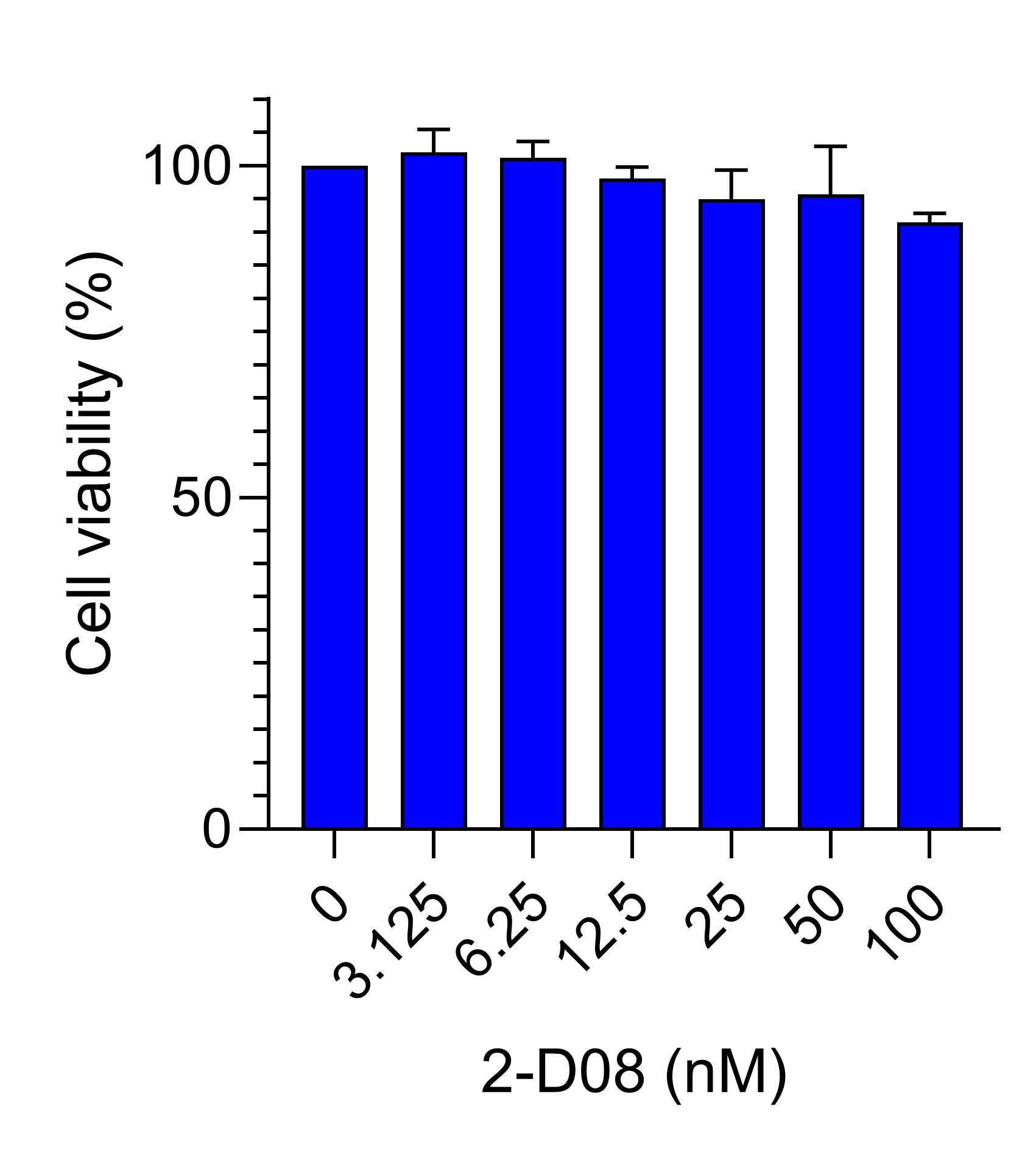

Supplement: Supplementary Figure 1 — Cytotoxic effect of Ubc9 inhibitor (2-D08) in A. aegypti CCL-125 cells. A. aegypti CCL-125 cells were incubated with culture medium containing Ubc9 inhibitor (2-D08) as indicated for 2 days. Attached cells were measured by cell viability assay. [file Image_1.tif]
